# Reframing Ankle Sprain Management: The Role of Thermography in Ligament Injury Monitoring

**DOI:** 10.3390/jcm15010134

**Published:** 2025-12-24

**Authors:** Victor-Luis Escamilla-Galindo, Daniel Fernández-Muñoz, Javier Fernández-Carmona, Julio A. Ceniza-Villacastín, Ismael Fernández-Cuevas

**Affiliations:** 1 Department of Nutrition and Sports Sciences, Universidad de La Rioja, 26006 Logroño, Spain; 2 Department of Research, ThermoHuman, 28030 Madrid, Spain; daniel.fernandez@thermohuman.com (D.F.-M.); julioalfonso.ceniza@alumno.ucjc.edu (J.A.C.-V.);; 3 Department of Performance, Watford FC, London AL2 1BZ, UK; javier.fernandez@watfordfc.com; 4 Strength Training and Neuromuscular Performance Research Group, Faculty of Health Sciences—HM Hospitals, University Camilo José Cela, 28692 Madrid, Spain; 5 Department of Performance, Real Madrid CF, 28055 Madrid, Spain; 6 Sports Department, Faculty of Sciences for Physical Activity and Sport (INEF), Universidad Politécnica de Madrid, 28040 Madrid, Spain

**Keywords:** temperature, management, ankle sprain, sport injury

## Abstract

**Background**: Ankle sprains are one of the most frequent ligament injuries in elite sports. Despite their high incidence, current rehabilitation approaches are often based on time-based criteria and neglect the physiological status of the injured tissues. Infrared thermography (IRT) is a non-invasive tool useful for detecting temperature asymmetries related to inflammation and tissue dysfunction. This study aimed to analyze the temporal evolution of ankle temperature asymmetry during return-to-play (RTP). **Methods:** A retrospective observational study of 26 ankle injuries analyzed with thermography that met the inclusion criteria. Thermograms were processed with a software to calculate temperature asymmetry in the ankle region of interest (ankleROI). Statistical analyses included paired and one-sample t-tests, as well as linear regression models, to assess temporal changes throughout the RTP process. **Results**: A significant hyperthermic response was observed immediately after injury (Δ = +0.594 °C; *p* < 0.001, Cohen’s d = 0.918). The first significant asymmetry reduction occurred between 21.5 and 28.5 days post-injury (Δ = −0.488 °C; *p* = 0.004), with a consistent weekly decrease of −0.109 °C (95% CI [−0.143, −0.078]). These findings indicate a progressive decrease in decrement on thermal asymmetry over approximately four weeks of RTP. **Conclusions**: IRT demonstrates potential as a physiological monitoring tool during the RTP process after ankle sprains. The observed pattern of temperature recovery provides objective reference thresholds that could complement existing functional and clinical criteria.

## 1. Introduction

### 1.1. Ankle Sprains in Elite Sport

Ankle sprains represent one of the most prevalent ligament injuries in professional sports, particularly in football, where they account for up to 18% of all injuries and predominantly affect the ankle ligament complex [1]. The limited mechanical tolerance of the ankle joint ligaments and the stochastic movements of sports contribute to their high incidence of injury [2]. Regardless of their high incidence, ankle sprains are often underestimated and treated with a time-based management approach with an average return to play (RTP) of less than 15 days, leading to recurrence rates as high as 41% [1,3]. This underestimation and accelerated RTP contribute to high reinjury rates, chronic ankle instability, and long-term functional limitations [4].

Current rehabilitation strategies have been shown to poorly reflect the mechanisms through which the injury occurs. A systematic review by Wagemans et al. [3] found that most prescribed exercises for ankle sprains are basic, uniplanar, and do not incorporate functional demands such as single-limb support, multiplanar movement, or jumps, movements associated with the inciting events of ankle sprains in sports.

Recent efforts have emphasized the need for evidence-based, criteria-driven rehabilitation protocols that integrate standardized functional tests [5], clinical evaluations [6], and patient-reported outcome measures (e.g., the Ankle Function Score) to guide a safe and progressive return to sport [7]. While these approaches represent an important advance, they largely focus on functional recovery and movement and do not directly address the physiological state of injured tissues during RTP.

### 1.2. The Role of Physiological Monitoring in Return-to-Sport Decisions

The scientific literature has advanced in generating practical guidelines for treating ankle sprains, although recent quality assessments performed using the AGREE II tool reveal significant limitations. Bsoul et al. [8] concluded that none of the five high-quality guidelines analyzed in that review provided concrete physiological monitoring tools to guide return-to-play decisions, an omission of increasing concern given the high rate of recurrent ankle sprains and chronic ankle instability.

Some physiological monitoring tools during the RTP phases are based on clinical tests such as Magnetic Resonance Imaging to assess tissue condition, but these are slow and expensive, and imaging abnormalities can persist long after functional recovery [9,10]. Other tools, such as monitoring lactate levels or muscle damage using biochemical markers like creatine kinase (CK), are invasive [11].

In this sense, infrared thermography (IRT) has gained interest as a rapid, noninvasive imaging technique that uses skin temperature asymmetries associated with inflammatory, metabolic, and potential injury processes [12,13].

Previous studies have shown that IRT can identify meaningful thermal asymmetries in joint regions such as the knee during anterior cruciate ligament (ACL) rehabilitation, correlating with tissue recovery and guiding the progression between RTP stages [14]. These findings suggest that IRT could serve as a physiological criterion to detect tissue dysfunction, inflammation, or imbalance, thus enhancing the safety of decision-making regarding the return to sport [14,15].

### 1.3. Rationale for Applying IRT in Ankle Sprain Management

Although IRT has been applied in the context of major ligament injuries such as ACL tears, its use in monitoring the recovery process after ankle sprains remains unexplored. Due to the high recurrence rates and limited physiologic assessment provided by current assessment protocols in the treatment of ankle injuries, the integration of IRT could be a strategy for assessing tissue from a physiological perspective.

Also, monitoring thermal asymmetries throughout the recovery process may help to detect unresolved inflammation, incomplete ligament healing, or compensatory patterns, which would provide an objective criterion for physicians and performance staff working with athletes.

### 1.4. Aim of the Study

This study aims to analyze the application of IRT as a physiological monitoring tool during the rehabilitation process following ankle sprains in athletes. Specifically, it seeks to evaluate how thermal asymmetry measurements behave throughout the RTP period in athletes in order to have a framework for successful recovery and to have physiological criteria for making RTP decisions.

## 2. Materials and Methods

### 2.1. Study Design and Dataset

This was a retrospective observational study using thermographic data collected using ThermoHuman software, version 2.10 (Pema Thermo Group, Madrid, Spain). The dataset included 302 athletes registered with ankle injuries, all from various sports disciplines who sustained the injury during training or competition. Only unilateral injuries were considered; bilateral cases were excluded. All included injuries had been evaluated at least three times following the initial injury event.

The descriptive data of the sample, covering the assessments prior to injury and extending to the last available measurement for each athlete, are available in Appendix A.

### 2.2. Image Acquisition Protocol

Thermographic images were obtained by trained technicians specialized in the ThermoHuman method, using FLIR infrared cameras (Teledyne FLIR, Wilsonville, OR, USA) with a minimum thermal resolution of 320 × 240 pixels, fully compatible with the software application. All image acquisitions adhered to a standardized protocol for lower limb assessment, ensuring standardization in subject preparation, environmental conditions, camera distance, and body positioning. This positioning of the subjects has been established through expert consensus and standardized in previous studies [13,16]. Room temperature data was extracted from the image background temperature to ensure data consistency (*T Background* = 22.4 °C ± 2.06 °C). The athletes were instructed to avoid any type of training or treatment before the evaluation.

**Figure 1:**
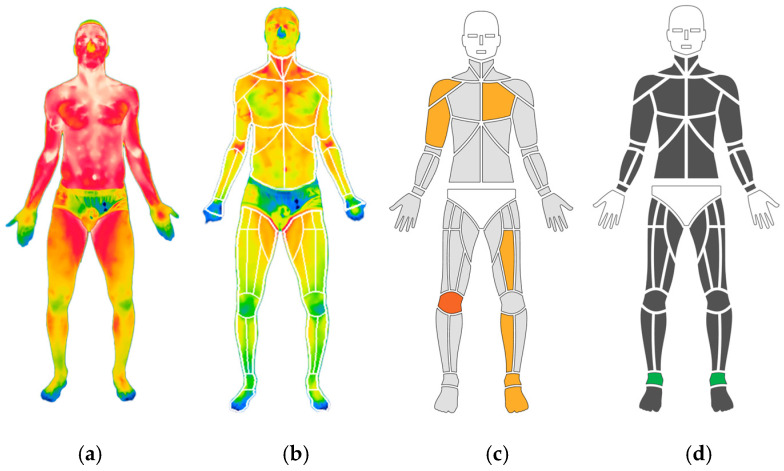
Segmentation of the anterior protocol to obtain the calculation of asymmetries of the regions of interest ((**a**) shows the raw thermogram; (**b**) the automatic segmentation of body regions; (**c**) the representation of asymmetries in an avatar including temperature data; and (**d**) displays the selected ROI in green for the analysis of an ankle sprain).

Each analysis was focused on the anterior part of the ankle, which was defined as the region of interest (ROI) for this study (Figure 1).

### 2.3. Data Processing and Quality Control

After the images were taken, they were downloaded into software, where automatic segmentation algorithms identified regions of interest (ROIs). From these, mean thermal asymmetry values were calculated by subtracting the temperature between the contralateral ankle ROIs (ankleROI).

To avoid potential variability due to different operators, devices, and settings, as described in the previous literature [16], a quality control process was implemented. One of the authors, an expert in thermographic analysis, manually reviewed all assessments to ensure correct segmentation and metadata accuracy. Cases with incomplete injury descriptions, poor image quality, or incorrect segmentation were excluded. An image was classified as being of poor quality if the focus of the foot boundaries was indistinct or blurred, which significantly increases the temperature variability. Furthermore, incorrect segmentation was defined if the segmentation failed to encompass the entire anatomical area or if more than 0.3% of the ankleROI boundary extended into the background of the image.

After this filtering process, 26 ankle injury cases met all inclusion criteria and were retained for statistical analysis.

### 2.4. Inclusion and Exclusion Criteria

Inclusion criteria:

- Unilateral ankle injury related to sports activity.
- Athlete evaluated in a sporting environment by a professional.
- At least three thermographic assessments post-injury, with the first within 20 days.

Exclusion criteria:

- Bilateral injuries.
- Absence of follow-up thermographic assessments.
- Inadequate image segmentation or missing clinical metadata.

### 2.5. Limitations of Data Collection Context

Because the thermographic recordings were collected under real-world conditions by different certified professionals in various sports environments, some factors such as ambient temperature, acclimatization time, and lighting could not be standardized across all cases. This limitation was partially mitigated by the rigorous post hoc image validation process described above.

### 2.6. Statistical Analysis

All statistical analyses were performed in order to assess thermal asymmetry patterns across the different time points relative to the injury.

For this purpose, a paired one-sided Student’s t-test was applied with the objective of determining whether the ankleROI presented changes in the acute phase compared with pre-injury levels in the entire database. In addition to *p*-values, we reported the mean paired difference, 95% confidence intervals (CI), and effect sizes using Cohen’s d (with thresholds of 0.2 = small, 0.5 = medium, 0.8 = large) to estimate the precision of the mean differences.

To evaluate the longitudinal change in thermal asymmetry, the baseline reference was established as the mean temperature asymmetry within the first 0.5–7.5 days post-injury. For each subsequent 7-day interval, the delta (difference from baseline) was computed. One-sided one-sample t-tests assessed whether the mean delta was significantly lower than zero.

To quantify the rate of change over time, linear regression models were fitted individually for each injury using post-injury data to estimate slopes of daily temperature asymmetry change. The average slope across injuries was obtained, and 95% bootstrap confidence intervals were computed. Daily slopes were reported until 70 days.

All statistical calculations were performed using Python 3.12 and visualizations were generated with Matplotlib library. The differences between mean values were considered statistically significant at *p* < 0.05.

## 3. Results

### 3.1. Hyperthermic Response in the Acute Phase of Injury

The analyses revealed a statistically significant difference between the pre-injury evaluations and the assessment following the injury event. The ankleROI exhibited a significant mean temperature increase of 0.594 °C (t = 4.683, *p* < 0.001, 95% CI [0.333, 0.856]). The effect size was large (Cohen’s d = 0.918), indicating a robust hyperthermic response immediately after injury.

### 3.2. Progress of Skin Temperature in the AnkleROI During Rehabilitation

Table 1 summarizes the post-injury time windows, ranging from the first week after injury to four months after the event.

The results indicate that the first significant reduction in temperature relative to the initial post-injury evaluation occurred during the interval of 21.5–28.5 days post-injury (mean Δ = −0.488 °C, 95% CI [−0.835, −0.142], *p* = 0.0049). Subsequent significant decreases were also observed in the intervals 28.5–35.5 days (Δ = −0.421 °C, *p* = 0.0010) and 42.5–49.5 days (Δ = −0.287 °C, *p* = 0.0190), confirming a sustained reduction in thermal asymmetry over time.

**Figure 2:**
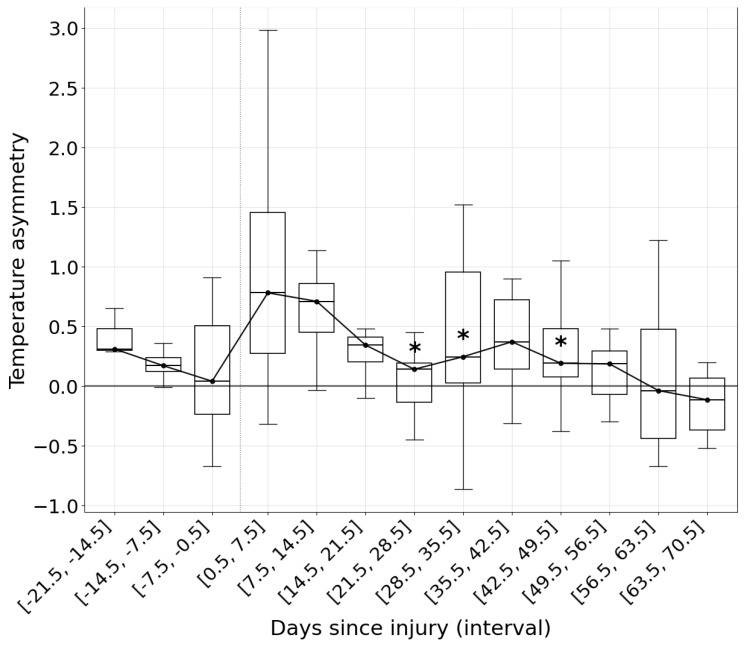
Evolution of AnkleROI temperature asymmetries during pre-injury weeks and the Return to Play (RTP) process. Data are displayed across weekly time windows (negative values on the X-axis represent pre-injury weeks and positive values represent the RTP process). (*) *Indicates a significant reduction in asymmetry temperature compared with the first post-injury week* (*p* < 0.05).

These results establish a reference framework for monitoring the physiological evolution of ankle temperature during rehabilitation (Figure 2). Within this framework, values are expected to align closely with the median, using the positive and negative standard deviation as upper and lower thresholds.

The average slope of change across injuries indicated a significant daily decrease of −0.0156 °C (95% CI [−0.0204, −0.0111]), equivalent to a weekly reduction of −0.1095 °C (95% CI [−0.1427, −0.0776]). The global linear model was a linear fit: y = −0.0156t + 0.8720 (R^2^ = 0.160).

**Figure 3:**
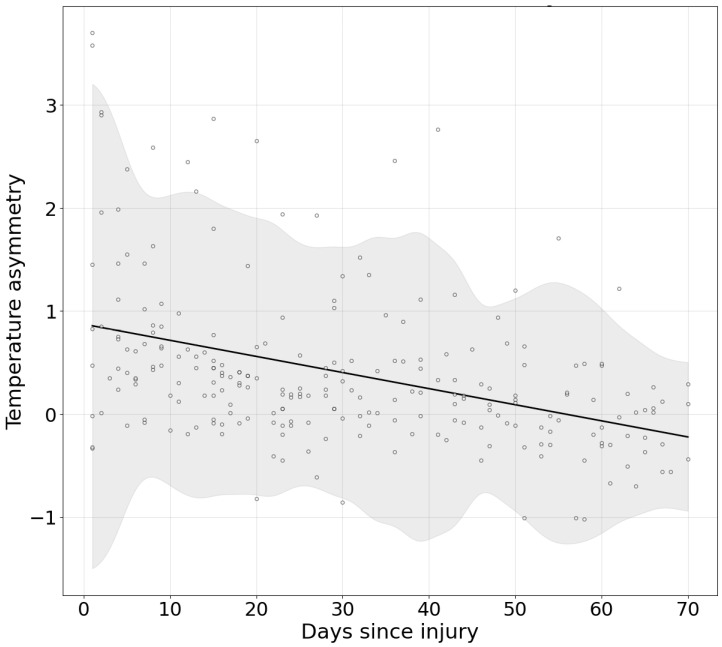
Linear regression model of daily ankleROI temperature asymmetry reduction during the RTP process. The gray area signifies the confidence interval for the data adjusted to the 95% CI, and the points represent the region’s assessments at different times during the RTP process.

Figure 3 illustrates the decrease in temperature during the recovery process, modeled according to the daily reduction estimated by the linear regression formula.

Overall, the results showed a significant increase in thermal asymmetry with a hyperthermic pattern in the ankleROI during the acute phase, followed by a gradual decrease in temperature over the following weeks. The first statistically significant reduction from baseline was detected approximately three weeks after the injury. Linear regression analyses also indicated a significant negative slope, confirming a consistent decrease in thermal asymmetry over time.

## 4. Discussion

### 4.1. Control of the Acute Response to the Injury

The temperature asymmetry metric between regions has been extensively studied in large population cohorts, showing that healthy individuals exhibit an exceptional degree of thermal balance, that is, minimal asymmetry when assessed [17,18].

The response of ankleROI asymmetries after injury, when compared with pre-injury data, demonstrated a significant increase in temperature. This rise is likely related to the systemic response triggered by ligamentous tissue damage [19]. The inflammatory processes, together with the biological repair mechanisms initiated during the acute phase of injury, may account for the observed temperature elevation in the region [20,21]. As reported in previous studies, ligamentous tissue injury is typically associated with a marked hyperthermic response [12,22,23,24]. This acute phase and the consequent hyperthermia can be monitored not only to provide information on the extent of the initial increase, supporting diagnostic considerations, but also as a reference point for subsequent monitoring throughout the RTP phases. An increase exceeding 0.59 °C may be associated with a poorer initial prognosis.

### 4.2. Monitoring Ankle Sprain RTP with Objective Data

The progression of skin temperature as an objective physiological parameter in sports injuries has gained increasing interest due to its relationship with tissue status and the rapid acquisition and analysis of the data [14]. To the authors’ knowledge, this is the first study to longitudinally evaluate temperature changes until return to competition in athletes. Previous studies using thermography have focused only on single time points in which the injury exhibited a hyperthermic response [22,24].

Ioannou, S. et al. [24] analyzed a case study of an amateur athlete over four assessments conducted within 42 days, focusing exclusively on absolute temperature values without considering inter-regional differences. The main finding was a hyperthermic response in the injured area, followed by a progressive decrease in absolute temperature, with a total reduction of 2.1 °C by the final assessment. However, analyzing absolute temperatures presents a limitation due to the variability introduced by the thermal camera across different captures. To improve data consistency, thermal asymmetries are preferred, as they are analyzed within each image, thereby minimizing the influence of sensor variability [25,26].

Current ankle RTP guidelines employ various clearance criteria to determine phase transitions and to assess whether an athlete can increase training load. One study proposed a rehabilitation algorithm to evaluate athlete status and progression throughout the RTP phases [27]. This algorithm defined four levels: from the physiotherapy perspective, assessment relied on clinical subjective examination, while from the strength and conditioning (S&C) perspective, it included balance tests (Y-balance test; modified stork balance test), endurance tests (heel-rise test), and jump ability tests (side hop test; triple hop test; square hop test; crossover hop for distance; 6 m timed crossover hop test). Once all levels were completed, the final RTP phase included sprinting, change in direction, and the Illinois test [27]. Importantly, none of these criteria incorporated a direct assessment of tissue status. A more recent systematic review on ankle rehabilitation guidelines reported that only 36.7% of them established monitoring criteria during the injury process, highlighting this as an area for improvement [8].

The present study monitored athletes throughout their RTP progression until full return to competition by analyzing ankleROI asymmetries. This approach allowed the development of a framework for assessing whether temperature evolution follows a normal or abnormal pattern during rehabilitation (Figure 2). In this sense, median values over time and their corresponding standard deviations may serve as reference thresholds for acceptable variability in ankleROI temperature (Table 1).

The temporal process of RTP in ankle sprains has been extensively investigated, and previous studies emphasize that both severity and location of the injury must be considered during rehabilitation. For example, Calder, J. et al. [28] reported that football players with grade II syndesmotic sprains with stable ankles on clinical testing returned to sport in approximately 45 days, whereas those with unstable ankles required about 64 days. In contrast, Flore, Z. et al. [1] observed a mean return of 29.9 days for lateral sprains of all grades. Finally, a meta-analysis showed that return-to-sport times differed according to clinical management: 39 days for conservatively treated cases and 71 days for surgically treated sprains [29].

In the present study, the temporal windows analyzed demonstrated that more than three weeks were required before significant decreases in ankle ROI temperature were observed (Table 1). In the fourth week (21.5 to 28.5 days after injury), skin temperature decreased by approximately 0.48 °C (*p* = 0.04), marking a physiological milestone related to the ligament healing process, marking the shift from a phase dominated by inflammation and proliferation to a phase of remodeling and maturation [30,31]. Benani et al. [30] highlight that the mechanical load produced by exercise plays a critical role during this period because it stimulates fibroblasts, leading to the production of a more organized extracellular matrix. Concurrently, other studies indicate that these fibroblasts become structurally more competent [31]. The fifth week also showed a significant reduction compared with initial post-injury values (−0.42 °C, *p* = 0.01), reinforcing thermography as a valid monitoring tool for tissue status. Interestingly, week six displayed an increase in temperature compared with the previous weeks, losing statistical significance relative to baseline values. Similar findings have been reported in ACL injury monitoring during phase transitions or with workload increments [14]. Such fluctuations may reflect acute structural responses to increased training load or incorporation into training on a regular basis, where demands are more variable compared to the controlled progression of rehabilitation [32]. These data highlight the non-linearity of recovery processes, in which reactive episodes may occur [33]. The subsequent weeks demonstrated renewed reductions in temperature (−0.28 °C, *p* = 0.01), ultimately reaching asymmetry values within the range considered physiologically acceptable between contralateral structures [17,34,35]. Due to the reduction in sample size in the final weeks, the data obtained during this period must be considered exploratory in nature and subject to a high degree of uncertainty.

The progressive reduction in ankleROI asymmetry, together with deviation thresholds, may serve as an objective physiological criterion for guiding the RTP process (Figure 3). A weekly asymmetry decrease of 0.10 °C (95% CI [−0.1427, −0.0776]) could represent a reference value within clinical decision-making, while also considering that the ankle region may react to workload increments.

This study has some limitations. The sample was heterogeneous in terms of injury types, due to the anonymous nature of the data, demographic information is unknown and could be a confounding factor in the analysis. Subdividing the data into groups could have been a confounding factor in the study, but with such a small sample size for some types of injury, it could have generated statistical noise, so it was not performed. Only biological time to return to competition was available, and no correlation was possible with other clinical or performance markers due to the different professionals conducting the RTP. Rehabilitation protocols also varied depending on each professional. Furthermore, the thermography technician had access solely to anonymized datasets, including the type of injury and temperature values per assessment day, without clinical details. As described in the methodology, potential sources of bias were minimized through rigorous checking of segmentation of thermographic images.

Despite these limitations, the authors consider that ankleROI temperature asymmetry data provide valuable objective information on ligament tissue status and its response to workload throughout the RTP process.

The integration of IRT into the daily routine of high-performance sports teams demonstrates high feasibility due to its non-invasive nature, speed of data acquisition, and objective output, such as temperature asymmetry data [13]. However, challenges persist, primarily related to the requirement for standardized protocols (e.g., acclimatization time and environmental control) to ensure data reliability [16,26]. Furthermore, improving the knowledge transfer from science to practice so that practitioners in sports institutions can correctly interpret thermal patterns and apply asymmetry thresholds remains a key obstacle. For this reason, these results, which seek to explain models for analyzing temperature and its asymmetries during the recovery phases of an ankle sprain, are valuable.

Regarding cost-effectiveness of the integration, studies have shown that while the initial investment in a thermal camera is necessary, the long-term benefit of objective, early identification of risk and the consequent reduction in re-injury rates or time lost from competition outweighs the cost [36,37]. Thus, the ability to monitor physiological milestones, as demonstrated by the 0.48 °C decrease in week 4, allows for an evidence-based progression criterion, which potentially improves decision-making in the RTP process and could help reduce the costs associated with sports absences.

Future research should integrate these measures with functional criteria and clinical tests, especially during load reaction times with the aim of relating temperature responses to functional measurements. Analyze a larger database to stratify the type of injury and analyze each type separately. As well as investigating temperature changes in other regions associated with ankle sprain injury, such as structures that are compensating.

## 5. Conclusions

This study provides novel evidence on the use of IRT as an objective monitoring tool during the RTP process following ankle sprains in athletes. The healing dynamics during the early recovery stage of ligament injuries were assessed through thermal analysis. By longitudinally evaluating ankleROI temperature asymmetries, a framework was created to monitor physiological milestones throughout RTP, with significant reductions in skin temperature emerging from the fourth week post-injury. These findings suggest that temperature asymmetry can serve as a reliable marker of tissue recovery, complementing current functional and clinical clearance criteria.

## Figures and Tables

**Table 1:** Temperature variation in ankleROI during post-injury temporary windows in days until the last assessment of the athletes.

Time Window (Days)	*n*	Mean Δ	P (*t*-test)	95% CI
7.5–14.5	10	−0.063	0.336	[−0.391, 0.264]
14.5–21.5	13	−0.210	0.080	[−0.517, 0.096]
21.5–28.5	13	−0.488 *	0.004	[−0.835, −0.142]
28.5–35.5	11	−0.421 *	0.001	[−0.647, −0.196]
35.5–42.5	11	−0.364	0.097	[−0.947, 0.219]
42.5–49.5	7	−0.287 *	0.019	[−0.552, −0.022]
49.5–56.5	7	−0.347	0.129	[−1.028, 0.335]
56.5–63.5	7	−0.654	0.052	[−1.492, 0.185]
63.5–70.5	5	−0.838	0.066	[−2.073, 0.396]

* Decreases in ankleROI temperature for temporal space were statistically significant at *p* < 0.05.

## Data Availability

Appendix A has the entire database of each athlete.

## Abbreviations

The following abbreviations are used in this manuscript:

RTP	Return-to-play
CK	Creatin kinase
IRT	Infrared Thermography
ACL	Anterior cruciate ligament
ROI	Region of interest
AnkleROI	Region of interest of the ankle

## Appendix A

This section presents the 26 ankle sprain cases analyzed in the study, including the type of injury categorized according to the Orchard Sports Injury Classification System (OSICS) 2024 and the severity grade, as well as the affected side.

The descriptive data include the last evaluation prior to the injury (with the day indicated in parentheses) and the temperature asymmetry observed between regions (also shown in parentheses). Additionally, the table displays the progression of temperature asymmetries between sides throughout the injury recovery process, following the same format (first the day of evaluation in parentheses, followed by the corresponding temperature asymmetry value).

**Table A1:** Descriptive data from the 26 sprain cases, including the type of injury, pre-injury values, and post-injury temperature data segmented by exact days.

Subjects	Type of Injury	Side	Preinjury (Day)/(°C)	Post Injury (Day)/(°C)																											
Athlete 1	AJXX Grade II	right	(−3)/(0.51)	(1)/ (0.83)	(2)/ (1.68)	(22)/ (−0.41)	(31)/ (0.52)	(32)/ (−0.02)	(41)/ (−0.2)	(49)/ (0.69)																					
Athlete 2	AJDX Grade II	left	(−12)/(0.12)	(2)/ (−0.01)	(8)/ (−0.46)	(16)/ (−0.37)																									
Athlete 3	AJXX Grade I	right	(−12)/(0.36)	(1)/ (−0.33)	(7)/ (−0.08)	(15)/ (−0.05)	(22)/ (0.01)	(28)/ (0.18)	(56)/ (0.19)																						
Athlete 4	AJXX Grade II	right	(−1)/(0.05)	(8)/ (0.86)	(15)/ (0.77)	(36)/ (2.46)																									
Athlete 5	AJXX Grade II	right	(−48)/(0.43)	(2)/ (2,9)	(4)/ (1.11)	(6)/ (0.34)	(7)/ (1.02)	(8)/ (0.43)	(9)/ (0.47)	(11)/ (0.98)	(12)/ (0.63)	(15)/ (0.31)	(17)/ (0.36)																		
Athlete 6	AJXX Grade I	right	(−16)/(0.65)	(15)/ (0.37)	(20)/ (0.35)	(36)/ (0.14)	(43)/ (0.33)	(50)/ (0.18)																							
Athlete 7	AJDX Grade II	left	(−2)/(−0.04)	(10)/ (0.16)	(13)/ (0.13)	(14)/ (−0.18)	(15)/ (0.09)	(17)/ (−0.09)	(18)/ (0.09)	(22)/ (0.08)	(23)/ (−0.05)	(25)/ (−0.57)	(26)/ (0.08)	(28)/ (−0.24)	(33)/ (−0.02)	(34)/ (−0.01)	(46)/ (0.45)	(47)/ (0.31)	(50)/ (0.11)	(51)/ (0.32)	(53)/ (0.29)	(54)/ (0.3)	(55)/ (0.06)	(58)/ (0.45)	(60)/ (0.31)	(61)/ (0.3)	(63)/ (0.51)	(64)/ (−0.02)	(66)/ (−0.02)	(67)/ (−0.12)	(70)/ (−0.1)
Athlete 8	AJSX Grade II	left	(−6)/(0.6)	(7)/ (0.05)	(24)/ (−0.16)	(26)/ (0.36)	(27)/ (0.61)	(28)/ (0.24)	(36)/ (0.37)	(42)/ (0.25)	(43)/ (0.06)	(46)/ (0.13)	(47)/ (−0.09)	(49)/ (0.09)	(50)/ (−0.14)	(51)/ (1.01)	(54)/ (0.02)	(57)/ (1.01)	(59)/ (0.2)	(60)/ (0.28)	(62)/ (0.03)	(63)/ (0.21)	(66)/ (−0.06)								
Athlete 9	AJDX Grade III	left	(−61)/(0.6)	(4)/ (−0.24)	(5)/ (−0.4)	(11)/ (−0.12)	(15)/ (−0.45)	(16)/ (−0.23)	(18)/ (−0.41)	(19)/ (0.04)	(23)/ (−0.19)	(29)/ (−0.05)	(30)/ (0.04)	(36)/ (0.06)	(38)/ (0.19)	(44)/ (0.08)	(47)/ (−0.25)	(48)/ (0.01)	(61)/ (0.67)	(64)/ (0.7)	(65)/ (0.37)	(68)/ (0.56)	(70)/ (0.44)	(71)/ (1.05)							
Athlete 10	AJLX Grade II	right	(−1)/(−0.37)	(1)/ (−0.02)	(6)/ (0.29)	(10)/ (0.18)	(13)/ (0.56)	(14)/ (0.6)	(18)/ (0.3)	(24)/ (0.19)	(25)/ (0.2)	(31)/ (0.23)	(33)/ (−0.11)	(39)/ (0.44)	(42)/ (0.58)	(43)/ (0.19)	(56)/ (0.21)	(59)/ (0.14)	(60)/ (0.47)	(63)/ (0.2)	(65)/ (0.04)	(66)/ (0.26)	(70) (0.29)								
Athlete 11	AJXX Grade II	left	(−1)/(0.16)	(2)/ (−2.93)	(3)/ (−0.35)	(4)/ (−0.75)	(5)/ (0.11)	(6)/ (−0.61)																							
Athlete 12	AJLA Grade II	right	(−1)/(−0.15)	(4)/ (1.99)	(32)/ (1.52)	(37)/ (0.51)	(39)/ (−0.02)	(50)/ (1.2)	(51)/ (0.66)	(53)/ (−0.41)	(54)/ (−0.17)	(58)/ (−1.02)	(60)/ (−0.13)	(67)/ (−0.29)	(71)/ (−0.37)																
Athlete 13	AJDX Grade II	right	(−8)/(0.17)	(2)/ (0.85)	(18)/ (0.28)	(23)/ (0.05)	(25)/ (0.25)	(30)/ (0.32)	(32)/ (0.16)	(39)/ (0.21)	(44)/ (0.18)	(47)/ (0.04)	(53)/ (−0.13)	(57)/ (0.47)	(65)/ (−0.23)	(67)/ (−0.56)															
Athlete 14	AJXX Grade II	left	(−17)/ (−0.31)	(15)/ (−0.18)	(43)/ (−0.1)	(50)/ (−0.11)																									
Athlete 15	AJXX Grade II	left	(−1)/(−0.61)	(1)/ (−3.7)	(2)/ (−1.96)	(5)/ (−1.55)	(15)/ (−2.87)	(20)/ (−2.65)	(21)/ (−1.64)	(23)/ (−0.94)	(30)/ (−1.34)	(37)/ (−0.9)																			
Athlete 16	AJXX Grade II	right	(−46)/(0.31)	(9)/ (0.85)	(29)/ (1.03)	(29)/ (0.49)	(45)/ (0.63)																								
Athlete 17	AJXX Grade III	right	(−21)/(0.29)	(1)/ (1.45)	(41)/ (2.76)	(70)/ (1.11)																									
Athlete 18	AJXX Grade II	right	(−7)/(0.91)	(7)/ (1.46)	(13)/ (2.16)	(29)/ (1.1)	(33)/ (1.35)	(43)/ (1.16)	(48)/ (0.94)	(55)/ (1.71)	(62)/ (1.22)	(71)/ (1.67)																			
Athlete 19	AJLX Grade II	left	(−1)/(0.31)	(1)/ (−0.47)	(5)/ (−0.63)	(8)/ (−0.79)	(15)/ (−0.45)	(19)/ (−0.37)	(23)/ (0.45)																						
Athlete 20	AJLX Grade II	right	(−4)/(−0.67)	(1)/ (−0.32)	(16)/ (−0.19)	(20)/ (−0.82)	(23)/ (−0.2)	(30)/ (−0.86)																							
Athlete 21	AJXX Grade II	right	(−3)/(0.4)	(4)/ (1.46)	(8)/ (1.63)	(9)/ (0.64)																									
Athlete 22	AJMX Grade II	right	(−9)/(0.24)	(4)/ (0.44)	(17)/ (0.01)	(21)/ (0.69)	(28)/ (0.45)	(32)/ (−0.21)	(36)/ (0.52)	(38)/ (0.22)																					
Athlete 23	AJLX Grade II	left	(−14)/(0.01)	(6)/ (−0.35)	(7)/ (−0.68)	(12)/ (0.19)	(13)/ (0.45)	(15)/ (−0.52)	(16)/ (−0.48)	(19)/ (−0.26)	(20)/ (−0.65)	(23)/ (0.24)	(24)/ (0.11)	(26)/ (−0.18)	(28)/ (−0.37)	(29)/ (−0.5)	(35)/ (−0.96)	(39)/ (−1.11)	(41)/ (−0.33)												
Athlete 24	AJLA Grade II	left	(−2)/(0.1)	(9)/ (−1.07)	(11)/ (−0.56)	(16)/ (0.1)	(24)/ (0.07)	(29)/ (−0.05)																							
Athlete 25	AJXX grade II	left	(−4)/(−0.5)	(4)/ (−0.73)	(9)/ (−0.66)	(11)/ (−0.3)	(16)/ (−0.4)	(18)/ (−0.41)	(23)/ (0.11)	(25)/ (−0.17)	(30)/ (−0.42)	(34)/ (−0.42)	(39)/ (−0.53)	(44)/ (−0.15)	(46)/ (−0.29)	(51)/ (−0.48)	(58)/ (−0.49)	(60)/ (−0.49)													
Athlete 26	AJLX Grade II	right	(−3)/(0.67)	(1)/ (3.58)	(5)/ (2.38)	(8)/ (2.59)	(12)/ (2.45)	(15)/ (1.8)	(19)/ (1.44)	(23)/ (1.94)	(27)/ (1.93)																				
